# Intergenerational ‘transmission’ of intimate partner violence and abuse: interpretations and implications

**DOI:** 10.1016/j.lanepe.2025.101299

**Published:** 2025-04-09

**Authors:** Roxanne C. Keynejad, Louise M. Howard

**Affiliations:** King's Women's Mental Health, Institute of Psychiatry, Psychology and Neuroscience, King's College London, Dr Crespigny Park, London, SE5 8AF, UK

Intimate partner violence and abuse (IPVA) is a major public health problem; consequences include suicide, homicide, major injuries, sexually transmitted infections, gynaecological problems and mental health conditions.^1^ In the first European Union violence against women survey, lifetime IPVA prevalence was 51.7% for ever-partnered women: 48.5% for psychological, 20% for physical and 8.4% for sexual IPVA.^2^ Identification of modifiable determinants of IPVA is therefore urgently needed.

Parental IPVA increases the risk of IPVA in the next generation as victims/survivors or perpetrators but the extent of this risk is not clear. Herbert et al.^3^ used data from the Avon Longitudinal Study of Parents and Children (ALSPAC), a pregnancy cohort recruited in the 1990's, to prospectively study relationships between parental IPVA identified during pregnancy and subsequently, and IPVA in their children aged 18–21 years. Notably, even in this relatively affluent, educated sample, 37–39% of mothers reported IPVA, including 24–25% experiencing physical abuse and 7–8% controlling behaviour. As expected, the authors identified associations between maternal IPVA victimisation and subtypes of young adult IPVA, although many attenuated to the null after adjustment for prenatal maternal IPVA and socioeconomic factors. The strongest adjusted association was between maternal physical IPVA and IPVA perpetration by their young adult sons (relative risk: 1.45, 95% confidence interval: 1.05–2.00), suggesting that maternal physical IPVA could be a feasible target for interventions to reduce perpetration in subsequent generations. The study estimated that up to 10% of young adult IPVA perpetrators may be accounted for by prior maternal IPVA, with a large majority of them also experiencing childhood maltreatment.

As acknowledged by the authors, the study is limited by the cohort's higher than average socio-economic status, lack of ethnic diversity, disproportionate attrition of male children and those experiencing socio-economic adversity, and the use of non-validated IPVA questions to ask pregnant women about their partners' emotional and physical cruelty. In addition, moderators and mediators of these relationships were not examined. It should be noted that the concept of intergenerational ‘transmission’ should not be interpreted as implying direct causation, as the determinants of IPVA are complex and at multiple levels, with gender inequity an underlying driver.^1^

The findings underscore the importance of IPVA detection and response as early as possible in young women and children's lives. This includes opportunistic identification during routine healthcare contacts, such as reproductive health, primary care, maternity and paediatric healthcare.^4^ There is increasing evidence that such identification is feasible and can improve outcomes when practitioners are trained to safely ask and respond, while avoiding victim blaming.^1^ There is less evidence on the feasibility, safety and outcomes of opportunistic identification of potential IPVA perpetrators; rigorous multi-centre randomised controlled trials of identification and interventions to address IPVA perpetration are required. Furthermore, public health responses include the need for sustained, sufficient funding for IPVA specialist services to support survivors and their children.

Herbert et al.^3^'s study will be of interest in the UK, whose government has set an ambitious target of halving violence against women and girls (VAWG) in the next decade. The authors' findings, suggesting intergenerational ‘transmission’ of IPVA, albeit without establishing causation, support the need for investment in cross-sectoral evidence-based interventions across the life course. They also contribute to growing calls to unify efforts to end violence against both women and children, worldwide, given their close relationship.^5^ The WHO and UN Women^6^ ‘RESPECT women’ framework advocates prevention and education, with additional recognition of socio-economic drivers of VAWG, such as poverty, unsafe environments, and women's financial disempowerment. Although Herbert et al.^3^'s study was conducted in peace time, the prevalence of IPVA often increases during periods of armed conflict.^7^ Current global geopolitical instability means that future research must consider the impact of conflict, displacement and related stressors such as climate events and financial crises, on associations between parental and child IPVA.

Growing international focus on young people's mental and physical health should drive research into interventions addressing potential mediators of the association between parental and youth IPVA, such as substance misuse, lack of economic participation, and traumatic stress. Interventions should target multiple levels of the socio-ecological framework, such as systemic barriers to gender equity at the community level and norms around substance use at the meso-social level. Gender-transformative interventions aiming to challenge patriarchal norms show promise,^8^^,^^9^ but require sustained investment, trained, supported staff and engagement of pan-societal stakeholders.^1^ However, misogynistic online content countering pro-social narratives evolves rapidly,^10^ requiring research to innovate and prioritise public health and policy making approaches, such as online controls and minimum alcohol unit pricing.

Herbert et al.^3^'s study demonstrates, encouragingly, that exposure to parental IPVA does not render future IPVA victimisation or perpetration inevitable. Greater research is required into protective factors, moderators and mediators of the association, and effective interventions across the life course, to break the cycle of violence so detrimental to health and well-being.

## Contributors

After discussion, Dr Keynejad wrote the first draft and Prof Howard edited the draft. Both authors then agreed the final version.

## Declaration of interests

Both authors declare no conflicts of interest.
